# Supplementary material for: CyMIRA: The Cytonuclear Molecular Interactions Reference for Arabidopsis
Source: Genome Biol Evol. 2019 Jul 8;11(8):2194–202. doi: 10.1093/gbe/evz144 (PMC6685490; doi:10.1093/gbe/evz144)
Supplement: evz144_Supplementary_Data [file evz144_supplementary_data.zip › Supplementary_Material.pdf]

Supplementary Text

The following summarizes the databases used to generate the CyMIRA classification, including our methods in converting the information in each database into the calls reported in CyMIRA.

eSLDB

The eukaryotic Subcellular Localization DataBase provides up-to-date subcellular predictions of entire proteomes for six eukaryotic species (*A. thaliana*, *Caenorhabditis elegans*, *Drosophila melanogaster*, *Homo sapiens*, *Mus musculus*, and *Saccharomyces cerevisiae*) based on experimental evidence when available, homology searches to closely related species with experimental evidence, and *in silico* predictions (Pierleoni, et al. 2007). We identified all *A. thaliana* proteins annotated as being targeted to the mitochondria or plastids, and proteins with targeting predictions to both organelles were treated as dual targeted. All other *A. thaliana* proteins that were described in eSLDB, but not marked as targeted to the mitochondria or plastids, were labeled as targeted to "Other". In total, eSLDB indicated that 847, 4424, and 69 are targeted to the mitochondria, plastids, or both, respectively, with 13170 proteins that are targeted to other subcellular localizations.

LocDB

LocDB is a protein subcellular localization database that catalogs experimental and *in silico* protein localization information from published studies for both *H. sapiens* and *A. thaliana* (Rastogi and Rost 2011). We downloaded all database entries from *A. thaliana* that were localized to the mitochondria or plastids. Protein entries that occurred in both datasets were treated as dual targeted. All other *A. thaliana* proteins that were described in LocDB, but not localized to the mitochondria or plastids were labeled as targeted to "Other". In total, LocDB indicated that 446, 1527, and 234 are targeted to the mitochondria, plastids, or both, respectively, with 1792 proteins that are targeted to other subcellular localizations.

Organelle DB

Organelle DB is a protein localization database that serves as a repository of experimental subcellular localization data for more than 138 organisms, including *A. thaliana* and *Oryza sativa* (Wiwatwattana, et al. 2007). We downloaded all *A. thaliana* entries that were associated with the following localizations: mitochondria – mitochondrion, nucleoid, organelle inner membrane, proton-transporting ATP synthase complex, respiratory chain complex I-IV, ribonucleoprotein complex (mitochondrial), succinate dehydrogenase complex (ubiquinone); plastids – endopeptidase Clp complex, nucleoid, organelle inner membrane, proton-transporting ATP synthase complex , ribonucleoprotein complex (plastid), thylakoid, thylakoid membrane. All other *A. thaliana* proteins that were described in Organelle DB, but not localized to the mitochondria or plastids were labeled as targeted to "Other". In total, Organelle DB indicated that 512, 276, and 11 are targeted to the mitochondria, plastids, or both, respectively, with 2032 proteins that are targeted to other subcellular localizations.

PA GOSUB

Proteome Analyst: Gene Ontology Molecular Function and Subcellular Localization v3.0 is a database with protein subcellular localizations for entire proteomes of a number of organisms that span the tree of life (Lu, et al. 2005). For each protein, PA GOSUB provides a protein

targeting prediction score and a non-targeted prediction score for 10 different subcellular compartments (chloroplast, cytoplasm, endoplasmic reticulum, extracellular, golgi, mitochondrion, nucleus, peroxisome, plasma membrane, and vacuole). We identified all proteins with mitochondrial-targeted prediction scores larger than not-mitochondrial-targeted prediction scores and all proteins with chloroplast-targeted prediction scores larger than not-chloroplast-targeted prediction scores as mitochondrial and plastid localized, respectively. Proteins for which both mitochondrial- and plastid-targeted prediction scores were greater than their associated not-targeted prediction scores were treated as dual localized. All other *A. thaliana* proteins that were described in PA GOSUB, but not marked as targeted to the mitochondria or plastids, were labeled as targeted to "Other". In total, PA GOSUB indicated that 985, 730, and 14 are targeted to the mitochondria, plastids, or both, respectively, with 11094 proteins that are targeted to other subcellular localizations.

PPDB

The Plant Proteome Database is a curated and up-to-date database featuring experimentally validated protein subcellular localization information for both *A. thaliana* and *Zea mays* (Sun, et al. 2009). We queried the database for all proteins with curated localizations in *A. thaliana* mitochondria or plastids. Genes with curated localizations to both mitochondria and plastids were treated as dual-targeted. All other *A. thaliana* proteins that were described in PPDB, but not localized to the mitochondria or plastids were labeled as targeted to "Other". In total, PPDB indicated that 327, 1570, and 73 are localized to the mitochondria, plastids, or both, respectively, with 1172 proteins that are targeted to other subcellular localizations.

TAIR

The *Arabidopsis* Information Resource is a database that collects, curates, and reviews detailed information about *A. thaliana* genes, including subcellular targeting predictions (Reiser, et al. 2017). We downloaded targeting prediction for all *A. thaliana* proteins and identified all entries that were predicted to be targeted to the mitochondria or plastids. Proteins with targeting predictions to both mitochondria and plastids were treated as dual-targeted. All other *A. thaliana* proteins that had associated targeting prediction, but not targeted to the mitochondria or plastids were labeled as targeted to "Other". In total, TAIR indicated that 397, 1598, and 266 are targeted to the mitochondria, plastids, or both, respectively, with 3402 proteins that are targeted to other subcellular localizations.

SWISS PROT

We downloaded all 15,859 entries for *A. thaliana* in the manually annotated and reviewed UniProt-KB/Swiss-Prot database (Poux, et al. 2017) and obtained subcellular localization information for each protein. We identified all proteins with curated subcellular localizations of either the mitochondria or chloroplast, and proteins with both organelles listed were treated as dual-targeted. All other *A. thaliana* proteins with accompanying subcellular localization information that were not targeted to the mitochondria or plastids were labeled as targeted to "Other". In total, SWISS-PROT indicated that 311, 657, and 20 are targeted to the mitochondria, plastids, or both, respectively, with 4773 proteins that are targeted to other subcellular localizations.

SUBA

The Subcellular Localisation Database for *Arabidopsis* proteins provides comprehensive and up-to-date subcellular localization based on both experimental validation and using a suite of predictive algorithms (Hooper, et al. 2017). We decided to split the database into two components: experimentally validated proteins (SUBA-experimental), and *in silico* subcellular localization predictions (SUBA-predicted). Experimental data from SUBA is validated with fluorescently tagged proteins or mass spectrometry. We obtained experimentally validated subcellular localization inferences by querying all *A. thaliana* proteins localized to either the mitochondria or the chloroplast, and proteins that were inferred to be localized to both organelles were treated as dual-targeted. Other *A. thaliana* proteins with experimentally determined localizations beside the mitochondria or plastids were labeled as targeted to “Other”. For subcellular localization predictions, we queried all *A. thaliana* proteins predicted by SUBAcon, a consensus of 22 different predictive tools, to be localized to the mitochondria, the plastids, or both. Other *A. thaliana* proteins with SUBAcon predictions, but not predicted to be targeted to the mitochondria or plastids, were labeled as targeted to “Other”. In total, SUBA-experimental inferred that 1217, 2128, and 785 proteins are localized to the mitochondria, plastids, or both, respectively, with 6369 proteins that are targeted to other subcellular localizations. SUBA-predicted predicted that 2369, 2644, and 97 proteins would be targeted to the mitochondria, plastids, or both, respectively, with 21469 proteins that are targeted to other subcellular localizations.

Supplementary Tables

Table S1. Summary of the number of gene assignments that are shared by each possible subset of databases in the sense of a Venn diagram. For example, there are eight *A. thaliana* genes that are predicted to be targeted to the mitochondrial by LocDB and Organelle DB but by no other databases.

Database Subset	Mitochondria	Plastid	Dual	Other
eSLDB	712	0	0	1954
LocDB	11	0	0	3
LocDB eSLDB	1	0	0	5
LocDB Organelle DB	8	0	0	0
LocDB Organelle DB eSLDB	1	0	0	0
LocDB Organelle DB PA_GOSUB PPDB SUBA_exp SUBA_pred SWISS_PROT TAIR	23	0	0	2
LocDB Organelle DB PA_GOSUB PPDB SUBA_exp SUBA_pred SWISS_PROT TAIR eSLDB	1	18	0	1
LocDB Organelle DB PA_GOSUB PPDB SUBA_exp SUBA_pred TAIR	32	0	0	29
LocDB Organelle DB PA_GOSUB PPDB SUBA_exp SUBA_pred TAIR eSLDB	1	17	0	16
LocDB Organelle DB PA_GOSUB PPDB SUBA_exp SWISS_PROT TAIR	1	0	0	8
LocDB Organelle DB PA_GOSUB PPDB SUBA_pred SWISS_PROT TAIR	17	0	0	21
LocDB Organelle DB PA_GOSUB PPDB SUBA_pred SWISS_PROT TAIR eSLDB	2	0	0	0
LocDB Organelle DB PA_GOSUB PPDB SUBA_pred TAIR	1	0	0	1
LocDB Organelle DB PA_GOSUB PPDB TAIR	1	0	0	1
LocDB Organelle DB PA_GOSUB SUBA_exp	1	0	0	2
LocDB Organelle DB PA_GOSUB SUBA_exp SUBA_pred	22	0	0	2
LocDB Organelle DB PA_GOSUB SUBA_exp SUBA_pred SWISS_PROT	28	0	0	7
LocDB Organelle DB PA_GOSUB SUBA_exp SUBA_pred SWISS_PROT eSLDB	2	0	0	6
LocDB Organelle DB PA_GOSUB SUBA_pred	1	0	0	3
LocDB Organelle DB PA_GOSUB SUBA_pred eSLDB	0	3	0	5
LocDB Organelle DB PA_GOSUB SUBA_pred SWISS_PROT	4	0	0	2
LocDB Organelle DB PA_GOSUB SUBA_pred SWISS_PROT eSLDB	1	0	0	0
LocDB Organelle DB PPDB SUBA_exp SUBA_pred SWISS_PROT TAIR	7	102	0	0
LocDB Organelle DB PPDB SUBA_exp SUBA_pred TAIR	21	0	0	0
LocDB Organelle DB PPDB SUBA_exp SWISS_PROT TAIR	1	0	0	4
LocDB Organelle DB PPDB SUBA_pred SWISS_PROT TAIR	1	0	0	16
LocDB Organelle DB PPDB SUBA_pred TAIR	4	102	0	15
LocDB Organelle DB SUBA_exp	33	0	0	65
LocDB Organelle DB SUBA_exp eSLDB	3	0	0	79
LocDB Organelle DB SUBA_exp SUBA_pred	50	0	0	11
LocDB Organelle DB SUBA_exp SUBA_pred eSLDB	5	0	0	26
LocDB Organelle DB SUBA_exp SUBA_pred SWISS_PROT	1	1	0	1
LocDB Organelle DB SUBA_pred	5	7	0	2
LocDB Organelle DB SUBA_pred SWISS_PROT	1	0	0	2
LocDB PA_GOSUB	7	0	0	1
LocDB PA_GOSUB PPDB SUBA_exp SUBA_pred SWISS_PROT TAIR	10	0	0	1
LocDB PA_GOSUB PPDB SUBA_exp SUBA_pred SWISS_PROT TAIR eSLDB	0	95	0	0
LocDB PA_GOSUB PPDB SUBA_exp SUBA_pred TAIR	10	0	0	2
LocDB PA_GOSUB PPDB SUBA_pred TAIR	1	0	0	5
LocDB PA_GOSUB PPDB SUBA_pred TAIR eSLDB	0	146	0	2
LocDB PA_GOSUB SUBA_exp	3	0	0	20
LocDB PA_GOSUB SUBA_exp SUBA_pred	11	0	0	27
LocDB PA_GOSUB SUBA_exp SUBA_pred SWISS_PROT	21	0	0	8
LocDB PA_GOSUB SUBA_exp SUBA_pred SWISS_PROT eSLDB	0	12	0	2
LocDB PA_GOSUB SUBA_exp SWISS_PROT	1	0	0	1
LocDB PA_GOSUB SUBA_pred	3	0	0	1
LocDB PA_GOSUB SUBA_pred eSLDB	0	223	1	1
LocDB PA_GOSUB SWISS_PROT	1	0	0	4
LocDB PPDB SUBA_exp SUBA_pred SWISS_PROT TAIR	5	331	6	12
LocDB PPDB SUBA_exp SUBA_pred TAIR	10	0	9	12
LocDB PPDB SUBA_exp SUBA_pred TAIR eSLDB	1	0	0	14
LocDB PPDB SUBA_exp TAIR	2	0	0	28
LocDB PPDB SUBA_pred TAIR	1	634	2	0
LocDB SUBA_exp	31	0	0	1

LocDB SUBA_exp eSLDB	1	0	0	2
LocDB SUBA_exp SUBA_pred	28	0	16	2
LocDB SUBA_exp SUBA_pred eSLDB	1	0	0	4
LocDB SUBA_exp SUBA_pred SWISS_PROT	4	47	1	1
LocDB SUBA_pred	2	906	62	1
LocDB SUBA_pred SWISS_PROT	1	0	0	5
Organelle DB	81	18	2	0
Organelle DB eSLDB	4	0	0	0
Organelle DB PA_GOSUB	23	0	0	0
Organelle DB PA_GOSUB eSLDB	1	5	0	1
Organelle DB PA_GOSUB PPDB SUBA_exp SUBA_pred TAIR	9	0	0	4
Organelle DB PA_GOSUB PPDB SUBA_exp SWISS_PROT TAIR eSLDB	0	1	0	30
Organelle DB PA_GOSUB PPDB SUBA_exp TAIR	1	0	0	12
Organelle DB PA_GOSUB PPDB SUBA_pred SWISS_PROT TAIR	18	0	0	7
Organelle DB PA_GOSUB PPDB SUBA_pred SWISS_PROT TAIR eSLDB	3	0	0	5
Organelle DB PA_GOSUB PPDB SUBA_pred TAIR	13	0	0	1
Organelle DB PA_GOSUB PPDB SWISS_PROT TAIR	1	0	0	1
Organelle DB PA_GOSUB PPDB TAIR eSLDB	0	1	0	1
Organelle DB PA_GOSUB SUBA_exp	4	0	0	0
Organelle DB PA_GOSUB SUBA_exp SUBA_pred	13	0	0	3
Organelle DB PA_GOSUB SUBA_exp SUBA_pred eSLDB	1	0	0	5
Organelle DB PA_GOSUB SUBA_exp SUBA_pred SWISS_PROT	6	0	0	1
Organelle DB PA_GOSUB SUBA_pred	10	0	0	3
Organelle DB PA_GOSUB SUBA_pred SWISS_PROT	4	0	0	1
Organelle DB PPDB SUBA_exp SUBA_pred SWISS_PROT TAIR	1	0	0	1
Organelle DB PPDB SUBA_exp SUBA_pred TAIR	6	0	0	1
Organelle DB PPDB SUBA_exp SWISS_PROT TAIR	0	1	0	0
Organelle DB PPDB SUBA_exp TAIR	0	0	2	2
Organelle DB PPDB SUBA_pred SWISS_PROT TAIR	6	0	0	1
Organelle DB PPDB SUBA_pred TAIR	2	0	0	4
Organelle DB PPDB TAIR	1	0	0	2
Organelle DB SUBA_exp	4	0	7	64
Organelle DB SUBA_exp SUBA_pred	14	0	0	13
Organelle DB SUBA_exp SUBA_pred SWISS_PROT	2	0	0	12
Organelle DB SUBA_pred	4	0	0	13
Organelle DB SWISS_PROT	1	0	0	1
PA_GOSUB	274	0	0	2
PA_GOSUB eSLDB	17	3875	64	2
PA_GOSUB PPDB SUBA_exp SUBA_pred TAIR	8	0	0	2
PA_GOSUB PPDB SUBA_exp SUBA_pred TAIR eSLDB	1	0	0	1
PA_GOSUB PPDB SUBA_exp SWISS_PROT TAIR eSLDB	0	3	0	2
PA_GOSUB PPDB SUBA_pred SWISS_PROT TAIR	11	3	0	9
PA_GOSUB PPDB SUBA_pred TAIR	17	0	0	19
PA_GOSUB PPDB SWISS_PROT TAIR eSLDB	1	0	0	2
PA_GOSUB PPDB TAIR	2	0	0	3
PA_GOSUB PPDB TAIR eSLDB	0	24	0	1
PA_GOSUB SUBA_exp	6	0	0	1
PA_GOSUB SUBA_exp eSLDB	1	0	4	2
PA_GOSUB SUBA_exp SUBA_pred	71	0	0	1
PA_GOSUB SUBA_exp SUBA_pred eSLDB	3	0	0	11
PA_GOSUB SUBA_exp SUBA_pred SWISS_PROT	22	0	0	14
PA_GOSUB SUBA_exp SWISS_PROT	1	0	0	53
PA_GOSUB SUBA_exp SWISS_PROT eSLDB	0	1	0	31
PA_GOSUB SUBA_pred	176	0	0	140
PA_GOSUB SUBA_pred eSLDB	5	0	0	55
PA_GOSUB SUBA_pred SWISS_PROT	46	0	0	84
PA_GOSUB SUBA_pred SWISS_PROT eSLDB	2	0	0	98
PA_GOSUB SWISS_PROT	8	0	0	2
PPDB SUBA_exp SUBA_pred SWISS_PROT TAIR	3	12	0	2
PPDB SUBA_exp SUBA_pred TAIR	28	0	0	1
PPDB SUBA_exp SUBA_pred TAIR eSLDB	3	0	0	3
PPDB SUBA_exp SWISS_PROT TAIR	0	0	7	8
PPDB SUBA_exp TAIR	2	0	29	2
PPDB SUBA_pred SWISS_PROT TAIR	6	0	0	4

PPDB SUBA_pred TAIR	20	0	0	6
PPDB SWISS_PROT TAIR	1	0	0	5
PPDB TAIR	10	83	18	20
SUBA_exp	338	0	702	14
SUBA_exp eSLDB	15	0	0	10
SUBA_exp SUBA_pred	265	0	0	9
SUBA_exp SUBA_pred eSLDB	9	0	0	0
SUBA_exp SUBA_pred SWISS_PROT	5	0	0	3
SUBA_exp SUBA_pred SWISS_PROT eSLDB	2	0	0	2
SUBA_exp SWISS_PROT	2	33	2	1
SUBA_pred	1156	0	0	1
SUBA_pred eSLDB	45	0	0	7
SUBA_pred SWISS_PROT	13	0	0	1
SUBA_pred SWISS_PROT eSLDB	1	0	0	15
SWISS_PROT	11	0	4	5
SWISS_PROT eSLDB	1	0	0	12
LocDB Organelle DB PA_GOSUB PPDB SUBA_exp SUBA_pred SWISS_PROT	0	0	0	13
LocDB Organelle DB PA_GOSUB PPDB SUBA_exp SUBA_pred SWISS_PROT eSLDB	0	0	0	1
LocDB Organelle DB PA_GOSUB PPDB SUBA_exp SWISS_PROT TAIR eSLDB	0	0	0	1
LocDB Organelle DB PA_GOSUB PPDB SUBA_pred	0	0	0	1
LocDB Organelle DB PA_GOSUB PPDB SUBA_pred SWISS_PROT	0	0	0	3
LocDB Organelle DB PA_GOSUB PPDB SUBA_pred SWISS_PROT eSLDB	0	0	0	1
LocDB Organelle DB PA_GOSUB PPDB SUBA_pred TAIR eSLDB	0	0	0	3
LocDB Organelle DB PA_GOSUB PPDB SWISS_PROT TAIR	0	0	0	1
LocDB Organelle DB PA_GOSUB SUBA_exp SUBA_pred eSLDB	0	0	0	2
LocDB Organelle DB PA_GOSUB SUBA_exp SUBA_pred SWISS_PROT TAIR	0	0	0	2
LocDB Organelle DB PA_GOSUB SUBA_exp SUBA_pred SWISS_PROT TAIR eSLDB	0	0	0	3
LocDB Organelle DB PA_GOSUB SUBA_exp SUBA_pred TAIR	0	0	0	16
LocDB Organelle DB PA_GOSUB SUBA_exp SUBA_pred TAIR eSLDB	0	0	0	12
LocDB Organelle DB PA_GOSUB SUBA_exp SWISS_PROT	0	0	0	30
LocDB Organelle DB PA_GOSUB SUBA_exp SWISS_PROT eSLDB	0	0	0	12
LocDB Organelle DB PA_GOSUB SUBA_exp SWISS_PROT TAIR	0	0	0	67
LocDB Organelle DB PA_GOSUB SUBA_exp TAIR	0	0	0	22
LocDB Organelle DB PA_GOSUB SUBA_exp TAIR eSLDB	0	0	0	113
LocDB Organelle DB PA_GOSUB SUBA_pred SWISS_PROT TAIR	0	0	0	85
LocDB Organelle DB PA_GOSUB SUBA_pred SWISS_PROT TAIR eSLDB	0	0	0	6
LocDB Organelle DB PA_GOSUB SUBA_pred TAIR eSLDB	0	0	0	11
LocDB Organelle DB PA_GOSUB SWISS_PROT eSLDB	0	0	0	6
LocDB Organelle DB PA_GOSUB SWISS_PROT TAIR	0	0	0	5
LocDB Organelle DB PA_GOSUB SWISS_PROT TAIR eSLDB	0	0	0	0
LocDB Organelle DB PPDB SUBA_exp SUBA_pred	0	0	0	4
LocDB Organelle DB PPDB SUBA_exp SUBA_pred eSLDB	0	0	0	6
LocDB Organelle DB PPDB SUBA_exp SUBA_pred SWISS_PROT TAIR eSLDB	0	0	0	7
LocDB Organelle DB PPDB SUBA_exp SUBA_pred TAIR eSLDB	0	0	0	6
LocDB Organelle DB PPDB SUBA_exp TAIR eSLDB	0	0	0	1
LocDB Organelle DB PPDB SUBA_pred	0	0	0	5
LocDB Organelle DB PPDB SUBA_pred eSLDB	0	0	0	86
LocDB Organelle DB PPDB SUBA_pred SWISS_PROT TAIR eSLDB	0	0	0	113
LocDB Organelle DB PPDB SUBA_pred TAIR eSLDB	0	0	0	11
LocDB Organelle DB SUBA_exp SUBA_pred SWISS_PROT TAIR	0	0	0	13
LocDB Organelle DB SUBA_exp SUBA_pred SWISS_PROT TAIR eSLDB	0	0	0	2
LocDB Organelle DB SUBA_exp SUBA_pred TAIR	0	0	0	1
LocDB Organelle DB SUBA_exp SUBA_pred TAIR eSLDB	0	0	0	3
LocDB Organelle DB SUBA_exp SWISS_PROT TAIR	0	0	0	5
LocDB Organelle DB SUBA_pred eSLDB	0	0	0	10
LocDB Organelle DB SUBA_pred SWISS_PROT TAIR	0	0	0	7
LocDB Organelle DB SUBA_pred SWISS_PROT TAIR eSLDB	0	0	0	12
LocDB Organelle DB SUBA_pred TAIR	0	0	0	20
LocDB Organelle DB SUBA_pred TAIR eSLDB	0	0	0	0
LocDB Organelle DB SWISS_PROT TAIR	0	0	0	1
LocDB Organelle DB TAIR	0	0	0	5
LocDB Organelle DB TAIR eSLDB	0	0	0	6
LocDB PA_GOSUB PPDB SUBA_exp SUBA_pred	0	0	0	17
LocDB PA_GOSUB PPDB SUBA_exp SUBA_pred eSLDB	0	0	0	16

LocDB PA_GOSUB PPDB SUBA_exp SUBA_pred SWISS_PROT	0	0	0	4
LocDB PA_GOSUB PPDB SUBA_exp SUBA_pred SWISS_PROT eSLDB	0	0	0	1
LocDB PA_GOSUB PPDB SUBA_exp SUBA_pred TAIR eSLDB	0	0	0	1
LocDB PA_GOSUB PPDB SUBA_exp SWISS_PROT TAIR	0	0	0	2
LocDB PA_GOSUB PPDB SUBA_exp SWISS_PROT TAIR eSLDB	0	0	0	2
LocDB PA_GOSUB PPDB SUBA_pred	0	0	0	0
LocDB PA_GOSUB PPDB SUBA_pred eSLDB	0	0	0	0
LocDB PA_GOSUB PPDB SUBA_pred SWISS_PROT	0	0	0	2
LocDB PA_GOSUB PPDB SUBA_pred SWISS_PROT eSLDB	0	0	0	2
LocDB PA_GOSUB PPDB SUBA_pred SWISS_PROT TAIR	0	0	0	25
LocDB PA_GOSUB PPDB SUBA_pred SWISS_PROT TAIR eSLDB	0	0	0	42
LocDB PA_GOSUB PPDB SWISS_PROT eSLDB	0	0	0	18
LocDB PA_GOSUB PPDB SWISS_PROT TAIR	0	0	0	26
LocDB PA_GOSUB SUBA_exp eSLDB	0	0	0	21
LocDB PA_GOSUB SUBA_exp SUBA_pred eSLDB	0	0	0	11
LocDB PA_GOSUB SUBA_exp SUBA_pred SWISS_PROT TAIR	0	0	0	37
LocDB PA_GOSUB SUBA_exp SUBA_pred SWISS_PROT TAIR eSLDB	0	0	0	58
LocDB PA_GOSUB SUBA_exp SUBA_pred TAIR	0	0	0	1
LocDB PA_GOSUB SUBA_exp SUBA_pred TAIR eSLDB	0	0	0	1
LocDB PA_GOSUB SUBA_exp SWISS_PROT TAIR	0	0	0	2
LocDB PA_GOSUB SUBA_exp SWISS_PROT TAIR eSLDB	0	0	0	4
LocDB PA_GOSUB SUBA_exp TAIR	0	0	0	54
LocDB PA_GOSUB SUBA_exp TAIR eSLDB	0	0	0	107
LocDB PA_GOSUB SUBA_pred SWISS_PROT	0	0	0	91
LocDB PA_GOSUB SUBA_pred SWISS_PROT eSLDB	0	0	0	153
LocDB PA_GOSUB SUBA_pred SWISS_PROT TAIR	0	0	0	5
LocDB PA_GOSUB SUBA_pred SWISS_PROT TAIR eSLDB	0	0	0	11
LocDB PA_GOSUB SUBA_pred TAIR	0	0	0	7
LocDB PA_GOSUB SUBA_pred TAIR eSLDB	0	0	0	41
LocDB PA_GOSUB SWISS_PROT TAIR eSLDB	0	0	0	5
LocDB PA_GOSUB TAIR	0	0	0	6
LocDB PA_GOSUB TAIR eSLDB	0	0	0	1
LocDB PPDB SUBA_exp SUBA_pred	0	0	0	2
LocDB PPDB SUBA_exp SUBA_pred eSLDB	0	0	0	2
LocDB PPDB SUBA_exp SUBA_pred SWISS_PROT eSLDB	0	0	0	1
LocDB PPDB SUBA_exp SUBA_pred SWISS_PROT TAIR eSLDB	0	0	0	1
LocDB PPDB SUBA_exp SWISS_PROT TAIR	0	0	0	1
LocDB PPDB SUBA_pred	0	0	0	1
LocDB PPDB SUBA_pred eSLDB	0	0	0	3
LocDB PPDB SUBA_pred SWISS_PROT TAIR	0	0	0	8
LocDB PPDB SUBA_pred SWISS_PROT TAIR eSLDB	0	0	0	4
LocDB PPDB SUBA_pred TAIR eSLDB	0	0	0	4
LocDB SUBA_exp SUBA_pred SWISS_PROT eSLDB	0	0	0	2
LocDB SUBA_exp SUBA_pred SWISS_PROT TAIR	0	0	0	3
LocDB SUBA_exp SUBA_pred SWISS_PROT TAIR eSLDB	0	0	0	15
LocDB SUBA_exp SUBA_pred TAIR	0	0	0	22
LocDB SUBA_exp SUBA_pred TAIR eSLDB	0	0	0	1
LocDB SUBA_exp TAIR	0	0	0	0
LocDB SUBA_exp TAIR eSLDB	0	0	0	1
LocDB SUBA_pred eSLDB	0	0	0	11
LocDB SUBA_pred SWISS_PROT TAIR	0	0	0	17
LocDB SUBA_pred SWISS_PROT TAIR eSLDB	0	0	0	6
LocDB SUBA_pred TAIR	0	0	0	6
LocDB SUBA_pred TAIR eSLDB	0	0	0	1
LocDB SWISS_PROT TAIR	0	0	0	4
LocDB TAIR eSLDB	0	0	0	1
Organelle DB PA_GOSUB PPDB SUBA_exp SUBA_pred	0	0	0	6
Organelle DB PA_GOSUB PPDB SUBA_exp SUBA_pred eSLDB	0	0	0	1
Organelle DB PA_GOSUB PPDB SUBA_exp SUBA_pred SWISS_PROT	0	0	0	0
Organelle DB PA_GOSUB PPDB SUBA_exp SUBA_pred SWISS_PROT eSLDB	0	0	0	0
Organelle DB PA_GOSUB PPDB SUBA_exp SUBA_pred SWISS_PROT TAIR	0	0	0	1
Organelle DB PA_GOSUB PPDB SUBA_exp SUBA_pred SWISS_PROT TAIR eSLDB	0	0	0	14
Organelle DB PA_GOSUB PPDB SUBA_exp SUBA_pred TAIR eSLDB	0	0	0	27
Organelle DB PA_GOSUB PPDB SUBA_pred	0	0	0	8

Organelle DB PA_GOSUB PPDB SUBA_pred eSLDB	0	0	0	6
Organelle DB PA_GOSUB PPDB SUBA_pred SWISS_PROT	0	0	0	7
Organelle DB PA_GOSUB PPDB SUBA_pred SWISS_PROT eSLDB	0	0	0	5
Organelle DB PA_GOSUB PPDB SUBA_pred TAIR eSLDB	0	0	0	27
Organelle DB PA_GOSUB PPDB SWISS_PROT	0	0	0	27
Organelle DB PA_GOSUB SUBA_exp eSLDB	0	0	0	1
Organelle DB PA_GOSUB SUBA_exp SUBA_pred SWISS_PROT eSLDB	0	0	0	1
Organelle DB PA_GOSUB SUBA_exp SUBA_pred SWISS_PROT TAIR	0	0	0	34
Organelle DB PA_GOSUB SUBA_exp SUBA_pred SWISS_PROT TAIR eSLDB	0	0	0	59
Organelle DB PA_GOSUB SUBA_exp SUBA_pred TAIR	0	0	0	13
Organelle DB PA_GOSUB SUBA_exp SUBA_pred TAIR eSLDB	0	0	0	17
Organelle DB PA_GOSUB SUBA_exp SWISS_PROT	0	0	0	1
Organelle DB PA_GOSUB SUBA_exp SWISS_PROT TAIR eSLDB	0	0	0	14
Organelle DB PA_GOSUB SUBA_exp TAIR	0	0	0	10
Organelle DB PA_GOSUB SUBA_exp TAIR eSLDB	0	0	0	2
Organelle DB PA_GOSUB SUBA_pred eSLDB	0	0	0	1
Organelle DB PA_GOSUB SUBA_pred SWISS_PROT eSLDB	0	0	0	3
Organelle DB PA_GOSUB SUBA_pred SWISS_PROT TAIR	0	0	0	353
Organelle DB PA_GOSUB SUBA_pred SWISS_PROT TAIR eSLDB	0	0	0	542
Organelle DB PA_GOSUB SUBA_pred TAIR	0	0	0	1
Organelle DB PA_GOSUB SUBA_pred TAIR eSLDB	0	0	0	2
Organelle DB PA_GOSUB SWISS_PROT	0	0	0	1
Organelle DB PA_GOSUB SWISS_PROT eSLDB	0	0	0	1
Organelle DB PA_GOSUB SWISS_PROT TAIR	0	0	0	24
Organelle DB PA_GOSUB SWISS_PROT TAIR eSLDB	0	0	0	32
Organelle DB PA_GOSUB TAIR	0	0	0	7
Organelle DB PA_GOSUB TAIR eSLDB	0	0	0	9
Organelle DB PPDB	0	0	0	21
Organelle DB PPDB eSLDB	0	0	0	9
Organelle DB PPDB SUBA_exp	0	0	0	26
Organelle DB PPDB SUBA_exp SUBA_pred	0	0	0	25
Organelle DB PPDB SUBA_exp SUBA_pred eSLDB	0	0	0	1
Organelle DB PPDB SUBA_exp SUBA_pred SWISS_PROT	0	0	0	1
Organelle DB PPDB SUBA_exp SUBA_pred SWISS_PROT eSLDB	0	0	0	2
Organelle DB PPDB SUBA_exp SUBA_pred SWISS_PROT TAIR eSLDB	0	0	0	18
Organelle DB PPDB SUBA_exp SUBA_pred TAIR eSLDB	0	0	0	36
Organelle DB PPDB SUBA_exp TAIR eSLDB	0	0	0	31
Organelle DB PPDB SUBA_pred	0	0	0	35
Organelle DB PPDB SUBA_pred eSLDB	0	0	0	7
Organelle DB PPDB SUBA_pred SWISS_PROT	0	0	0	7
Organelle DB PPDB SUBA_pred SWISS_PROT eSLDB	0	0	0	12
Organelle DB PPDB SUBA_pred SWISS_PROT TAIR eSLDB	0	0	0	11
Organelle DB PPDB SUBA_pred TAIR eSLDB	0	0	0	1
Organelle DB PPDB SWISS_PROT	0	0	0	1
Organelle DB SUBA_exp eSLDB	0	0	0	0
Organelle DB SUBA_exp SUBA_pred eSLDB	0	0	0	0
Organelle DB SUBA_exp SUBA_pred SWISS_PROT eSLDB	0	0	0	0
Organelle DB SUBA_exp SUBA_pred SWISS_PROT TAIR	0	0	0	18
Organelle DB SUBA_exp SUBA_pred SWISS_PROT TAIR eSLDB	0	0	0	22
Organelle DB SUBA_exp SUBA_pred TAIR	0	0	0	610
Organelle DB SUBA_exp SUBA_pred TAIR eSLDB	0	0	0	619
Organelle DB SUBA_exp TAIR	0	0	0	285
Organelle DB SUBA_exp TAIR eSLDB	0	0	0	155
Organelle DB SUBA_pred eSLDB	0	0	0	70
Organelle DB SUBA_pred SWISS_PROT	0	0	0	44
Organelle DB SUBA_pred SWISS_PROT eSLDB	0	0	0	211
Organelle DB SUBA_pred SWISS_PROT TAIR	0	0	0	148
Organelle DB SUBA_pred TAIR	0	0	0	7
Organelle DB SUBA_pred TAIR eSLDB	0	0	0	3
Organelle DB TAIR	0	0	0	2
Organelle DB TAIR eSLDB	0	0	0	1
PA_GOSUB PPDB	0	0	0	8
PA_GOSUB PPDB eSLDB	0	0	0	9
PA_GOSUB PPDB SUBA_exp	0	0	0	1822

PA_GOSUB PPDB SUBA_exp eSLDB	0	0	0	2194
PA_GOSUB PPDB SUBA_exp SUBA_pred	0	0	0	702
PA_GOSUB PPDB SUBA_exp SUBA_pred eSLDB	0	0	0	638
PA_GOSUB PPDB SUBA_exp SUBA_pred SWISS_PROT	0	0	0	57
PA_GOSUB PPDB SUBA_exp SUBA_pred SWISS_PROT eSLDB	0	0	0	51
PA_GOSUB PPDB SUBA_exp SUBA_pred SWISS_PROT TAIR	0	0	0	68
PA_GOSUB PPDB SUBA_exp SUBA_pred SWISS_PROT TAIR eSLDB	0	0	0	65
PA_GOSUB PPDB SUBA_exp SWISS_PROT	0	0	0	23
PA_GOSUB PPDB SUBA_exp TAIR	0	0	0	26
PA_GOSUB PPDB SUBA_pred	0	0	0	3
PA_GOSUB PPDB SUBA_pred eSLDB	0	0	0	4
PA_GOSUB PPDB SUBA_pred SWISS_PROT	0	0	0	4
PA_GOSUB PPDB SUBA_pred SWISS_PROT eSLDB	0	0	0	6
PA_GOSUB PPDB SUBA_pred SWISS_PROT TAIR eSLDB	0	0	0	7
PA_GOSUB PPDB SUBA_pred TAIR eSLDB	0	0	0	2
PA_GOSUB PPDB SWISS_PROT	0	0	0	18
PA_GOSUB PPDB SWISS_PROT eSLDB	0	0	0	16
PA_GOSUB SUBA_exp SUBA_pred SWISS_PROT eSLDB	0	0	0	5
PA_GOSUB SUBA_exp SUBA_pred SWISS_PROT TAIR	0	0	0	4
PA_GOSUB SUBA_exp SUBA_pred SWISS_PROT TAIR eSLDB	0	0	0	10
PA_GOSUB SUBA_exp SUBA_pred TAIR	0	0	0	5
PA_GOSUB SUBA_exp SUBA_pred TAIR eSLDB	0	0	0	31
PA_GOSUB SUBA_exp SWISS_PROT TAIR	0	0	0	17
PA_GOSUB SUBA_exp SWISS_PROT TAIR eSLDB	0	0	0	1
PA_GOSUB SUBA_exp TAIR	0	0	0	1
PA_GOSUB SUBA_exp TAIR eSLDB	0	0	0	0
PA_GOSUB SUBA_pred SWISS_PROT TAIR	0	0	0	3
PA_GOSUB SUBA_pred SWISS_PROT TAIR eSLDB	0	0	0	17
PA_GOSUB SUBA_pred TAIR	0	0	0	22
PA_GOSUB SUBA_pred TAIR eSLDB	0	0	0	3
PA_GOSUB SWISS_PROT eSLDB	0	0	0	6
PA_GOSUB SWISS_PROT TAIR	0	0	0	2
PA_GOSUB TAIR	0	0	0	10
PA_GOSUB TAIR eSLDB	0	0	0	12
PPDB	0	0	0	1
PPDB eSLDB	0	0	0	1
PPDB SUBA_exp eSLDB	0	0	0	1
PPDB SUBA_exp SUBA_pred	0	0	0	46
PPDB SUBA_exp SUBA_pred eSLDB	0	0	0	75
PPDB SUBA_exp SUBA_pred SWISS_PROT	0	0	0	615
PPDB SUBA_exp SUBA_pred SWISS_PROT eSLDB	0	0	0	549
PPDB SUBA_exp SUBA_pred SWISS_PROT TAIR eSLDB	0	0	0	143
PPDB SUBA_exp SWISS_PROT	0	0	0	50
PPDB SUBA_exp TAIR eSLDB	0	0	0	63
PPDB SUBA_pred	0	0	0	17
PPDB SUBA_pred eSLDB	0	0	0	197
PPDB SUBA_pred SWISS_PROT	0	0	0	118
PPDB SUBA_pred SWISS_PROT eSLDB	0	0	0	8
PPDB SUBA_pred TAIR eSLDB	0	0	0	2
PPDB TAIR eSLDB	0	0	0	2
SUBA_exp SUBA_pred SWISS_PROT TAIR	0	0	0	5
SUBA_exp SUBA_pred SWISS_PROT TAIR eSLDB	0	0	0	9
SUBA_exp SUBA_pred TAIR	0	0	0	4152
SUBA_exp SUBA_pred TAIR eSLDB	0	0	0	3650
SUBA_exp SWISS_PROT eSLDB	0	0	0	456
SUBA_exp SWISS_PROT TAIR	0	0	0	193
SUBA_exp TAIR	0	0	0	19
SUBA_exp TAIR eSLDB	0	0	0	7
SUBA_pred SWISS_PROT TAIR	0	0	0	57
SUBA_pred SWISS_PROT TAIR eSLDB	0	0	0	50
SUBA_pred TAIR	0	0	0	44
SUBA_pred TAIR eSLDB	0	0	0	12
SWISS_PROT TAIR	0	0	0	2
SWISS_PROT TAIR eSLDB	0	0	0	2

TAIR	0	0	0	5
TAIR eSLDB	0	0	0	10

Table S2. Full list of references used for manual curation of cytonuclear complexes

Category	References
**ACCase**	Konishi and Sasaki 1994; Sasaki and Nagano 2014; Rockenbach et al. 2016; Salie and Thelen 2016; Sudianto and Chaw 2019
**Chlororibosome**	Bonen and Calixte 2005; Tiller et al. 2012; Sloan et al. 2014; Bieri et al. 2017; Boerema et al. 2018
**Clp protease**	Nishimura et al. 2015; Nishimura and Wijk 2015; Rockenbach et al. 2016; Williams et al. 2019
**DNA-RRR**	Zaegel et al. 2006; Lamesch et al. 2012; Cupp and Nielsen 2014; Zhang et al. 2015; Gualberto and Newton 2017; Córdoba et al. 2019
**TAT complex**	Lamesch et al. 2012; Carrie et al. 2016
**Mitoribosome**	Bonen and Calixte 2005; Waltz et al. 2019
**OXPHOS**	Millar et al. 2004; Lu et al. 2005; Meyer et al. 2008; Klodmann et al. 2010; Klodmann et al. 2011; Lamesch et al. 2012; Senkler et al. 2017; Huang et al. 2019; Ligas et al. 2019
**Photosynthesis**	Kurisu et al. 2003; Friso et al. 2004; Nelson et al. 2004; Jensen et al. 2007; Izumi et al. 2012; Shikanai 2016; Bezouwen et al. 2017; Laughlin et al. 2019
**PPR**	Lurin et al. 2004; Toole et al. 2008; Fujii et al. 2010; Cheng et al. 2016
**Transcription and transcript maturation**	Hess and Borner 1999; Walter et al. 2002; Gu et al. 2003; Perrin et al. 2004; Kuhn et al. 2007; Schmidt von Braun et al. 2007; Yu et al. 2008; Canino et al. 2009; Delannoy et al. 2009; Chen et al. 2010; Falcon de Longevialle et al. 2010; Gobert et al. 2010; Placido et al. 2010; Richter et al. 2010; Babiychuk et al. 2011; Bryant et al. 2011; Gerdes et al. 2011; Sharwood et al. 2011; Apitz et al. 2014; Brown et al. 2014; Cohen et al. 2014; Schmitz-linneweber et al. 2015; Zhang et al. 2015; Stoll and Binder 2016; Wang et al. 2016; Shevtsov et al. 2018; http://seve.ibmp.unistra.fr/plantrna
**tRNA aminoacylation**	(Mireau et al. 1996; Akashi et al. 1998; Menand et al. 1998; Uwer et al. 1998; Souciet et al. 1999; Peeters et al. 2000; Duchene et al. 2001; Berg et al. 2005; Duchene et al. 2005; Pujol et al. 2007; Pujol et al. 2008; Hopper et al. 2011)

Supplementary References

Akashi K, Grandjean O, Small I. 1998. Potential dual targeting of an Arabidopsis archaebacterial-like histidyl-tRNA synthetase to mitochondria and chloroplasts 1. FEBS Lett. 431:39–44.

Apitz J, Schmied J, Lehmann MJ, Hedtke B, Grimm B. 2014. GluTR2 complements a hema1 mutant lacking glutamyl-tRNA reductase 1, but is differently regulated at the post-translational level. Plant Cell Physiol. 55:645–657.

Babiychuk E, Vandepoele K, Wissing J, Garcia-Diaz M, Rycke R De, Akbari H. 2011. Plastid gene expression and plant development require a plastidic protein of the mitochondrial transcription termination factor family. PNAS 108:6674–6679.

Berg M, Rogers R, Muralla R, Meinke D. 2005. Requirement of aminoacyl-tRNA synthetases for gametogenesis and embryo development in Arabidopsis. plan J.:866–878.

Bezouwen LS Van, Caffarri S, Kale RS, Kou R, Thunnissen AWH, Oostergetel GT, Boekema EJ. 2017. Subunit and chlorophyll organization of the plant photosystem II supercomplex. Nat. Plants 17080:1–11.

Bieri P, Leibundgut M, Saurer M, Boehringer D, Ban N. 2017. The complete structure of the chloroplast 70S ribosome in complex with translation factor pY. EMBO J. 36:475–486.

Boerema AP, Aibara S, Paul B, Tobiasson V, Kimanius D, Forsberg BO, Wallden K, Lindahl E, Amunts A. 2018. Structure of the chloroplast ribosome with chl-RRF and hibernation-promoting factor. Nat. Plants 4:212–217.

Bonen L, Calixte S. 2005. Comparative aqnalysis of bacterial-origin genes for plant mitochondrial ribosomal proteins. Mol Biol Evol 23:701–712.

Brown GG, Francs-small CC, Ostersetzer-biran O. 2014. Group II intron splicing factors in plant mitochondria. Front. Plant Sci. 5:1–13.

Bryant N, Lloyd J, Sweeney C, Myouga F, Meinke D. 2011. IDentification of nuclear genes encoding chloroplast-localized proteins required for embryo development in Arabidopsis. Plant Physiol. 155:1678–1689.

Canino G, Bocian E, Barbezier N, Echeverrı M, Forner J, Binder S, Marchfelder A. 2009. Arabidopsis encodes four tRNase Z enzymes. Plant Physiol. 150:1494–1502.

Carrie C, Weibenberger S, Soll J. 2016. Plant mitochondria contain the protein translocase subunits TatB and TatC. Co. Biol.:3935–3947.

Chen P, Jäger G, Zheng B. 2010. Transfer RNA modifications and genes for modifying enzymes in Arabidopsis thaliana. BMC Pl 10:10–201.

Cheng S, Gutmann B, Zhong X, Ye Y, Fisher MF, Bai F, Castleden I, Small I. 2016. Redefining the structural motifs that determine RNA binding and RNA editing by pentatricopeptide repeat proteins in land plants. plan J. 85:532–547.

Cohen S, Zmudjak M, Francs-small CC, Malik S, Shaya F, Keren I, Belausov E, Many Y, Brown GG, Small I, et al. 2014. nMAT4, a maturase factor required for nad1 pre-mRNA

processing and maturation , is essential for holocomplex I biogenesis in Arabidopsis mitochondria. plan J. 78:253–268.

Córdoba JP, Fassolari M, Marchetti F, Soto D, Pagnussat GC, Zabaleta E. 2019. Different Types of CA Domains Are Present in Complex I from Immature Seeds and Adult Plants in Arabidopsis thaliana. Japanese Soc. Plant Physiol.

Cupp JD, Nielsen BL. 2014. Mitochondrion Minireview : DNA replication in plant mitochondria. Mitochondrion 19:231–237.

Delannoy E, Le Ret M, Faivre-Nitschke E, Estavillo GM, Bergdoll M, Taylor NL, Pogson BJ, Small I, Imbault P, Gualberto JM. 2009. Arabidopsis tRNA adenosine deaminase arginine edits the wobble nucleotide of chloroplast tRNA Arg (ACG) and Is essential for efficient chloroplast translation. Plant Cell 21:2058–2071.

Duchene A-M, Giritch A, Hoffmann B, Lancelin D, Peeters NM, Duche A, Small ID, Zaepfel M, Mare L. 2005. Dual targeting is the rule for organellar aminoacyl-tRNA synthetases in Arabidopsis thaliana. PNAS 102:16484–16489.

Duchene A, Peeters N, Dietrich A, Cosset A, Small ID, Wintz H, Glyrs P. 2001. Overlapping destinations for two dual targeted glycyl-tRNA synthetases in arabidopsis thaliana and Phaseolus vulgaris. J. Biol. Chem. 276:15275–15283.

Falcon de Longevialle A, Small ID, Lurin C. 2010. Nuclearly encoded splicing factors implicated in RNA splicing in higher plant organelles. Mol. Plant 3:691–705.

Friso G, Giacomelli L, Ytterberg AJ, Peltier J, Rudella A, Sun Q, Wijk KJ Van. 2004. In-depth analysis of the thylakoid membrane proteome of Arabidopsis thaliana chloroplasts : new proteins, new functions, and a plastid proteome database. Plant Cell 16:478–499.

Fujii S, Bond CS, Small ID. 2010. Selection patterns on restorer-like genes reveal a conflict between nuclear and mitochondrial genomes throughout angiosperm evolution. PNAS 108:1723–1728.

Gerdes S, Yacoubi B El, Bailly M, Blaby IK, Blaby-haas CE, Jeanguenin L, Lara-núñez A, Pribat A, Waller JC, Wilke A, et al. 2011. Synergistic use of plant-prokaryote comparative genomics for functional annotations. BMC Genomics 12:1–16.

Gobert A, Gutmann B, Taschner A, Gobringer M, Holzmann J, Hartmann RK, Rossmanith W, Giegé P. 2010. A single Arabidopsis organellar protein has RNase P activity. Nat. Struct. Mol. Biol. 17:740–744.

Gu W, Jackman JE, Lohan AJ, Gray MW, Phizicky EM. 2003. tRNA His maturation: An essential yeast protein catalyzes addition of a guanine nucleotide to the 5’ end of tRNA His. Genes Dev. 17:2889–2901.

Gualberto JM, Newton KJ. 2017. Plant mitochondrial genomes: dynamics and mechanisms of mutation. Annu. Rev. Plant Biol. 68:225–252.

Hess WR, Borner T. 1999. Organellar RNA Polymerases of Higher Plants. Int. Rev. Cyrology 190:7674–7696.

Hooper CM, Castleden IR, Tanz SK, Aryamanesh N, Millar AH. 2017. SUBA4: the interactive data analysis centre for Arabidopsis subcellular protein locations. Nucleic Acids

Research 45:D1064-D1074

Hopper AK, Pai DA, Engelke DR. 2011. Cellular dynamics of tRNAs and their genes. Fed. Eur. Biochem. Soc. 584.

Huang S, Braun H-P, Gawryluk RMR, Millar AH. 2019. Mitochondrial complex II of plants: subunit composition, assembly and function in respiration and signaling. Plant J.

Izumi M, Tsunoda H, Suzuki Y, Makino A, Ishida H. 2012. RBCS1A and RBCS3B, two major members within the Arabidopsis RBCS multigene family, function to yield sufficient Rubisco content for leaf photosynthetic capacity. J. Exp. Bot. 63:2159–2170.

Jensen P, Bassi R, Boekema EJ, Dekker JP, Jansson S, Leister D, Robinson C, Vibe H. 2007. Structure, function and regulation of plant photosystem I. Biochim. Biophys. Acta 1767:335–352.

Klodmann J, Senkler M, Rode C, Braun H. 2011. Defining the protein complex proteome of plant mitochondria. Plant Physiol. 157:587–598.

Klodmann J, Sunderhaus S, Nimtz M, Jansch L, Braun HP. 2010. Internal architecture of mitochondrial complex I from Arabidopsis thaliana. Plant Cell 22:797–810.

Konishi T, Sasaki Y. 1994. Compartmentalization of two forms of acetyl-CoA carboxylase in plants and the origin of their tolerance toward herbicides. PNAS 91:3598–3601.

Kuhn K, Bohne A, Liere K, Weihe A, Bo T. 2007. Arabidopsis phage-type RNA polymerases : accurate in vitro transcription of organellar genes. Plant Cell 19:959–971.

Kurisu G, Zhang H, Smith JL, Cramer WA. 2003. Structure of the cytochrome b6f complex of oxygenic photosynthesis: tuning the cavity. Science (80-.). 302:1009–1015.

Lamesch P, Berardini TZ, Li D, Swarbreck D, Wilks C, Sasidharan R, Muller R, Dreher K, Alexander DL, Garcia-Hernandez M, et al. 2012. The Arabidopsis Information Resource (TAIR): Improved gene annotation and new tools. Nucleic Acids Res. 40:1202–1210.

Laughlin TG, Bayne AN, Trempe J, Savage DF, Davies KM. 2019. Structure of the complex I-like molecule NDH of oxygenic photosynthesis. Nature 566:411–417.

Ligas J, Pineau E, Bock R, Huynen MA, Meyer EH. 2019. The assembly pathway of complex I in Arabidopsis thaliana. Plant J.:447–459.

Lu P, Szafron D, Greiner R, Wishart DS, Fyshe A, Pearcy B, Poulin B, Eisner R, Ngo D, Lamb N. 2005. PA-GOSUB : a searchable database of model organism protein sequences with their predicted Gene Ontology molecular function and subcellular localization. Nucleic Acids Res. 33:147–153.

Lurin C, Andrés C, Aubourg S, Bellaoui M, Bitton F, Bruyère C, Caboche M, Debast C, Gualberto J, Hoffmann B, et al. 2004. Genome-wide analysis of arabidopsis pentatricopeptide repeat proteins reveals their essential role in organelle biogenesis. Plant Cell 16:2089–2103.

Menand B, Marechal L, Sakamoto W, Dietrich A, Wintz H. 1998. A single gene of chloroplast origin codes for mitochondrial and chloroplastic methionyl – tRNA synthetase in Arabidopsis thaliana. PNAS 95:11014–11019.

Meyer EH, Taylor NL, Millar AH. 2008. Resolving and identifying protein components of plant

mitochondrial respiratory complexes using three dimensions of gel electrophoresis. J. Proteome Res. 7:786–794.

Millar AH, Eubel H, Ja L, Kruft V, Heazlewood JL. 2004. Mitochondrial cytochrome c oxidase and succinate dehydrogenase complexes contain plant specific subunits. Plant Mol. Biol. 56:77–90.

Mireau H, Lancelin D, Small ID. 1996. The same arabidopsis gene encodes both cytosolic and mitochondrial alanyl-tRNA synthetases. Plant Cell 8:1027–1039.

Nelson N, Ben-shem A, Wise TGS. 2004. The complex architecture of oxygenic photosynthesis. Mol. Cell Biol. 5:1–13.

Nishimura K, Apitz J, Friso G, Kim J, Ponnala L, Grimm B, Wijk KJ Van. 2015. Discovery of a unique Clp component , ClpF , in chloroplasts: A proposed binary ClpF-ClpS1 adaptor complex functions in substrate recognition and delivery. Plant Cell 27:2677–2691.

Nishimura K, Wijk KJ Van. 2015. Organization, function and substrates of the essential Clp protease system in plastids. BBA - Bioenerg. 1847:915–930.

Peeters NM, Chapron A, Giritch A, Grandjean O, Lancelin D, Lhomme T, Vivrel A, Small I. 2000. Duplication and quadruplication of Arabidopsis thaliana cysteinyl- and asparaginyl-tRNA synthetase genes of organellar origin. J. Mol. Evol. 50:413–423.

Perrin R, Lange H, Grienenberger J, Gagliardi D. 2004. AtmtPNPase is required for multiple aspects of the 18S rRNA metabolism in Arabidopsis thaliana mitochondria. Nucleic Acids Res. 32:5174–5182.

Pierleoni A, Martelli PL, Fariselli P, Casadio R. 2007. eSLDB: eukaryotic subcellular localization database. Nucleic Acids Research 35:D208-212.

Placido A, Gobert A, Gallerani R, Mare L, Giege P. 2010. Plant mitochondria use two pathways for the biogenesis of tRNA His. Nucleic Acids Res. 38:7711–7717.

Poux S, Arighi CN, Magrane M, Bateman A, Wei CH, Lu Z, Boutet E, Bye AJH, Famiglietti ML, Roechert B, et al. 2017. On expert curation and scalability: UniProtKB/Swiss-Prot as a case study. Bioinformatics 33:3454-3460.

Pujol C, Bailly M, Kern D, Marechal L, Becker H, Duchene A. 2008. Dual-targeted tRNA-dependent amidotransferase ensures both mitochondrial and chloroplastic Gln-tRNA Gln synthesis in plants. PNAS 105:6481–6485.

Pujol C, Maréchal-drouard L, Duchêne A. 2007. How can organellar protein N-terminal sequences be dual targeting signals? In silico analysis and mutagenesis approach. J. Mol. Biol. 369:356–367.

Rastogi S, Rost B. 2011. LocDB: experimental annotations of localization for Homo sapiens and Arabidopsis thaliana. Nucleic Acids Research 39:D230-234.

Reiser L, Subramaniam S, Li D, Huala E. 2017. Using the Arabidopsis Information Resource (TAIR) to Find Information About Arabidopsis Genes. Curr Protoc Bioinformatics 60:1.11 1-45.

Richter U, Ku K, Okada S, Brennicke A, Weihe A, Bo T, Botanik M. 2010. A mitochondrial rRNA dimethyladenosine methyltransferase in Arabidopsis. Plant J. 1:558–569.

Rockenbach K, Havird JC, Grey Monroe J, Triant DA, Taylor DR, Sloan DB. 2016. Positive selection in rapidly evolving plastid-nuclear enzyme complexes. Genetics 204:1507–1522.

Salie MJ, Thelen JJ. 2016. Regulation and structure of the heteromeric acetyl-CoA carboxylase. Biochim. Biophys. Acta 1861:1207–1213.

Sasaki Y, Nagano Y. 2014. Plant acetyl-CoA carboxylase: structure, biosynthesis, regulation, and gene manipulation for plant breeding. Biosci. Biotechnol. Biochem. 8451:0916-8451.

Schmidt von Braun S, Sabetti A, Hanic-joyce PJ, Gu J, Schleiff E, Joyce PBM. 2007. Dual targeting of the tRNA nucleotidyltransferase in plants: not just the signal. J. Exp. Bot. 58:4083–4093.

Schmitz-linneweber C, Lampe M, Sultan LD, Ostersetzer-biran O. 2015. Organellar maturases: A window into the evolution of the spliceosome. BBA - Bioenerg. 1847:798–808.

Senkler J, Senkler M, Eubel H, Hildebrandt T, Lengwenus C, Schertl P, Wagner S, Wittig I, Braun H. 2017. The mitochondrial complexome of Arabidopsis thaliana. Plant J. 89:1079–1092.

Sharwood RE, Halpert M, Luro S, Schuster G, Stern DB. 2011. Chloroplast RNase J compensates for inefficient transcription termination by removal of antisense RNA. RNA 17:2165–2176.

Shevtsov S, Nevo-dinur K, Faigon L, Sultan LD, Zmudjak M, Markovits M, Ostersetzer-biran O. 2018. Control of organelle gene expression by the mitochondrial transcription termination factor mTERF22 in Arabidopsis thaliana plants. PLoS One:1–31.

Shikanai T. 2016. Chloroplast NDH : A different enzyme with a structure similar to that of respiratory NADH dehydrogenase. BBA - Bioenerg. 1857:1015–1022.

Sloan DB, Triant DA, Wu M, Taylor DR. 2014. Cytonuclear interactions and relaxed selection accelerate sequence evolution in organelle ribosomes. Mol. Biol. Evol. 31:673–682.

Souciet G, Menand B, Ovesna J, Cosset A, Dietrich A, Wintz H. 1999. Characterization of two bifunctional Arabdopsis thaliana genes coding for mitochondrial and cytosolic forms of valyl-tRNA synthetase and threonyl-tRNA synthetase by alternative use of two in-frame AUGs. FEBS 854:848–854.

Stoll B, Binder S. 2016. Two NYN domain containing putative nucleases are involved in transcript maturation in Arabidopsis mitochondria. Plant J. 85:278–288.

Sudianto E, Chaw S-M. 2019. Two independent plastid accD transfers to the nuclear genome of Gnetum and other insights on acetyl-CoA carboxylase evolution in gymnosperms. Genome Biol. Evol.:1–30.

Sun Q, Zybailov B, Majeran W, Friso G, Olinares PDB, van Wijk KJ. 2009. PPDB, the Plant Proteomics Database at Cornell. Nucleic Acids Research 37:D969-D974.

Tiller N, Weingartner M, Thiele W, Maximova E, Scho MA. 2012. The plastid-specific ribosomal proteins of Arabidopsis thaliana can be divided into non-essential proteins and genuine ribosomal proteins. plan J. 69:302–316.

Toole NO, Hattori M, Andres C, Iida K, Lurin C, Schmitz-linneweber C, Sugita M, Small I.

2008. On the expansion of the pentatricopeptide repeat gene family in plants. Mol. Biol. Evol. 25:1120–1128.

Uwer U, Willmitzer L, Altmann T. 1998. Inactivation of a glycyl-tRNA synthetase leads to an arrest in plant embryo development. Plant Cell 10:1277–1294.

Walter M, Kilian J, Kudla È. 2002. PNPase activity determines the efficiency of mRNA 3'-end processing, the degradation of tRNA and the extent of polyadenylation in chloroplasts. Eur. Mol. Biol. Organ. 21:6905–6914.

Waltz F, Nguyen TT, Arrivé M, Bochler A, Chicher J, Hammann P, Kuhn L, Quadrado M, Mireau H, Yashem Y, et al. 2019. Small is big in Arabidopsis mitochondrial ribosome. Nat. Plants 5:106–117.

Wang S, Bai G, Wang S, Yang L, Yang F, Wang Y. 2016. Chloroplast RNA-binding protein RBD1 promotes chilling tolerance through 23S rRNA processing in Arabidopsis. PLoS Genet. 1:1–21.

Williams AM, Friso G, Wijk KJ Van, Sloan DB. 2019. Extreme variation in rates of evolution in the plastid Clp protease complex. Plant J. 98:243-259.

Wiwatwattana N, Landau CM, Cope GJ, Harp GA, Kumar A. 2007. Organelle DB: an updated resource of eukaryotic protein localization and function. Nucleic Acids Research 35:D810-814.

Yu F, Liu X, Alsheikh M, Park S, Rodermel S. 2008. Mutations in SUPPRESSOR OF VARIEGATION1, a factor required for normal chloroplast translation, suppress var2-mediated leaf variegation in Arabidopsis. Plant Cell 20:1786–1804.

Zaegel V, Guermann B, Le Ret M, Andres C, Meyer D, Erhart M, Canaday J, Gualberto JM, Imbault P. 2006. The plant-specific ssDNA binding protein OSB1 Is involved in the stoichiometric transmission of mitochondrial DNA in Arabidopsis. Plant Cell 18:3548–3563.

Zhang J, Ruhlman TA, Sabir J, Blazier JC, Jansen RK. 2015. Coordinated rates of evolution between interacting plastid and nuclear genes in geraniaceae. Plant Cell 27:563–573.
